# Quantifying the phosphorylation timescales of receptor–ligand complexes: a Markovian matrix-analytic approach

**DOI:** 10.1098/rsob.180126

**Published:** 2018-09-19

**Authors:** M. López-García, M. Nowicka, C. Bendtsen, G. Lythe, S. Ponnambalam, C. Molina-París

**Affiliations:** 1 Department of Applied Mathematics, School of MathematicsSchool of Molecular and Cellular Biology, University of Leeds, LS2 9JT Leeds, UK; 2 Endothelial Cell Biology Unit, School of Molecular and Cellular Biology, University of Leeds, LS2 9JT Leeds, UK; 3 Department of Pathology and Cell Biology, Columbia University Medical Center, New York, NY, USA; 4 Quantitative Biology, Discovery Sciences, IMED Biotech Unit, AstraZeneca, Cambridge Science Park, Milton Road, CB4 0WG Cambridge, UK

**Keywords:** stochastic model, receptor–ligand interaction, phosphorylation, time to signalling threshold, quasi-birth-and-death process

## Abstract

Cells interact with the extracellular environment by means of receptor molecules on their surface. Receptors can bind different ligands, leading to the formation of receptor–ligand complexes. For a subset of receptors, called receptor tyrosine kinases, binding to ligand enables sequential phosphorylation of intra-cellular residues, which initiates a signalling cascade that regulates cellular function and fate. Most mathematical modelling approaches employed to analyse receptor signalling are deterministic, especially when studying scenarios of high ligand concentration or large receptor numbers. There exist, however, biological scenarios where low copy numbers of ligands and/or receptors need to be considered, or where signalling by a few bound receptor–ligand complexes is enough to initiate a cellular response. Under these conditions stochastic approaches are appropriate, and in fact, different attempts have been made in the literature to measure the timescales of receptor signalling initiation in receptor–ligand systems. However, these approaches have made use of numerical simulations or approximations, such as moment-closure techniques. In this paper, we study, from an analytical perspective, the stochastic times to reach a given signalling threshold for two receptor–ligand models. We identify this time as an extinction time for a conveniently defined auxiliary absorbing continuous time Markov process, since receptor–ligand association/dissociation events can be analysed in terms of quasi-birth-and-death processes. We implement algorithmic techniques to compute the different order moments of this time, as well as the steady-state probability distribution of the system. A novel feature of the approach introduced here is that it allows one to quantify the role played by each kinetic rate in the timescales of signal initiation, and in the steady-state probability distribution of the system. Finally, we illustrate our approach by carrying out numerical studies for the vascular endothelial growth factor and one of its receptors, the vascular endothelial growth factor receptor of human endothelial cells.

## 1. Introduction

Cells interact with the extracellular environment by means of molecules located on their surface, referred to as *receptors*. These receptors interact with extracellular molecules called *ligands*, so that bound receptor–ligand complexes are formed, which eventually phosphorylate, initiating downstream signalling in the cytoplasm, and leading to a cellular response. Phosphorylation of a particular class of receptors, receptor tyrosine kinases (RTKs), occurs upon sequential activation of tyrosine residues located in the intra-cellular tail of the receptors. In order to model cell behaviour regulated by receptor–ligand interactions, initial cell surface binding events and subsequent intra-cellular processes must be first quantified. Once this foundation is established, cellular behaviour can be analysed based on the number, state, and location of the molecules and complexes involved. The receptor population is involved in binding to the ligand, cross-linking to other receptors or membrane associated molecules, internalization, recycling, degradation and synthesis, broadly termed ‘trafficking’ events [1].

Detailed analyses of receptor–ligand interactions and phosphorylation kinetics on the cell membrane usually make use of mathematical models which ignore endocytosis (or internalization) events, and focus on the biochemical reactions taking place on the cell surface. For example, Starbuck *et al*. [2] consider a particular RTK, the epidermal growth factor receptor (EGFR), to study the role of epidermal growth factor (EGF) on mammalian fibroblasts. They argue that the receptor signal is generated at a rate proportional to the number of activated receptors present, so that the amount of phosphorylated ligand-bound dimeric complexes is directly related to the initiation of signalling cascades. Tan *et al*. [3] consider a mathematical model of pre-formed RTK receptor dimers, with instantaneous phosphorylation of ligand-bound dimeric complexes. However, phosphorylation is in fact a multi-step process, in which the different tyrosine domains of each receptor transfer phosphate (from ATP) onto side chains of specific tyrosine residues of the partner receptor, i.e. trans-autophosphorylation [4]. In Alarcón & Page [5], stochastic models of receptor oligomerization by a bivalent ligand are introduced to study the role of ligand-induced receptor cross-linking in cell activation. A particular feature of this study is that a small number of receptors is considered, making a stochastic approach more suitable than a deterministic one (see [6] for a comparison between deterministic and stochastic approaches for models of vascular endothelial growth factor receptors). In order to relate receptor–ligand dynamics on the cellular membrane to cell activation, the authors [5] introduce a threshold number of bound oligomers that need to be formed before a cellular response can take place. Once the stochastic process reaches this threshold, they study (by means of Gillespie simulations) the probability of staying above this threshold for a given time, *T* = 10 *k*^−1^_off_, which is identified with the time required for the activation of kinases and for the signalling pathway to be initiated [5].

In this study, we analyse receptor–ligand interactions and phosphorylation dynamics on the cell surface, to compute the time to reach a given signalling threshold [7], and the late time probability distribution of the system. To this end, we first introduce a mathematical model (instantaneous phosphorylation (IP) model), in which receptor monomers can bind a bivalent ligand, which allows a second receptor monomer to cross-link. This model is similar to Model 1 of Alarcón & Page [5]. However, rather than assuming that a fixed time interval above the threshold leads to a cellular response, we consider phosphorylation an intrinsic characteristic of the ligand cross-linked receptor dimers. In the IP model, ligand-bound receptor dimers are assumed to be instantaneously phosphorylated, so that the time to initiate the signalling cascade is identified with the time to reach a given threshold number of ligand-bound phosphorylated receptor dimers. This results in the analysis of a first-passage time or an absorption time in the theory of continuous time Markov processes. In the second model, the delayed phosphorylation (DP) model, phosphorylation of ligand-bound receptor dimers is considered as an additional reaction in the system, and we also consider the possibility of ligand-bound receptor dimer de-phosphorylation. We then compute the time to reach a given threshold number of phosphorylated ligand-bound receptor dimers in the DP model. Finally, the late time behaviour of the system is studied by analysing its stationary probability distribution.

As stated in Alarcón & Page [5], the analytical treatment of the multi-variate stochastic processes describing these biological receptor–ligand systems is typically extremely difficult, and numerical approaches, such as Gillespie simulations, are normally used instead. However, it is still possible to carry out an analytical study of these processes without the need to solve the corresponding master equation. Here, we do so by making use of a matrix-analytic technique and by considering a number of stochastic descriptors, conveniently defined in the spirit of Alarcón & Page [5]. This matrix-analytic approach, which has its origins in the seminal work by Neuts [8], allows us to study the stochastic descriptors of interest for moderate numbers of ligands and receptors in an *exact* way, as discussed in §2. Matrix-analytic techniques have historically been developed in the context of Queueing Theory [9]. However, more recently, they have been applied in Mathematical Biology [10–12].

We illustrate our methods by considering a receptor–ligand interaction involving vascular endothelial growth factors (VEGFs) and receptors (VEGFRs) in human endothelial cells. VEGFs are a family of bivalent ligands consisting of mammalian and virus-encoded members. The first member discovered was VEGF-A [13], which occurs in different isoforms of varying lengths. Mounting evidence suggests that the various isoforms are involved in diverse cellular responses [4]. VEGFs specifically bind to three type V RTKs, VEGFR1, VEGFR2 and VEGFR3, as well as co-receptors, such as neuropilins. In physiological conditions, the vascular endothelium expresses VEGFR1 and VEGFR2, whereas the lymphatic endothelium expresses VEGFR2 and VEGFR3 [14]. Each receptor has an extracellular domain for binding ligand, a trans-membrane domain and an intra-cellular or cytoplasmic domain [1]. Like many other RTKs, VEGFRs normally require dimerization to become activated: once VEGF binds to VEGFRs, the intra-cellular domains become activated by auto-phosphorylation and start cascades of intra-cellular enzymatic reactions [4]. We aim to develop a new quantitative study of receptor–ligand interaction and phosphorylation kinetics to aid our understanding of processes such as angiogenesis and vasculogenesis.

The paper is organized as follows. In §2, two different stochastic models are introduced to describe the association and dissociation dynamics of ligand-bound receptor monomers and dimers on the cell surface. The models include instantaneous phosphorylation or phosphorylation as an additional reaction. Matrix-analytic techniques are applied (for further details about these techniques, see appendices B and C) to study a number of stochastic descriptors of interest to the system, making use of an auxiliary absorbing continuous time Markov process. A particular feature of this method is that a sensitivity analysis (described in appendix D) to quantify the effect of association, dissociation and phosphorylation rates on the stochastic descriptors can be carried out. In §3.1, parameter estimation is carried out following arguments first described in Lauffenburger & Linderman [1], and applied to obtain the kinetic rates of the receptor–ligand system of interest (VEGFR2 and VEGF-A, respectively) from the physiological parameters given in §3.2. Finally, numerical results are presented in §3.3 and §3.4, followed by a discussion in §4. The notation used in the paper is introduced in appendix A.

## 2. Stochastic models

In this section, we introduce two different stochastic models for the interaction of a surface receptor and a bivalent ligand (see §3). The bivalent ligand can bind a receptor monomer, creating a bound monomeric complex. The free site of the ligand in a bound monomeric complex can then bind to a second receptor monomer, while these molecules diffuse on the cell surface. This leads to a bound dimeric complex, consisting of two receptors bound to a bivalent ligand.

In our models, receptor dimerization is ligand-induced, as the dimeric VEGF-A ligand binds and recruits two receptor monomers into a single complex (cross-linking). We thus assume that two monomeric and free receptors are not able to create a *pre-dimer* in the absence of ligand (ligand-induced dimerization or LID [15, LID model]). We note that the consideration of receptor pre-dimerization in the model does not significantly change the dynamics of the process, especially for low ligand concentrations [15], as considered here. In some instances, and for highly saturated situations, the existence of pre-dimers may alter the dynamics of the system (see, for example, MacGabhann & Popel [15, Figs. 2 and 3] for details). On the other hand, there is experimental support for the following hypothesis: free VEGFR2 is observed (electron microscopy) in monomeric form on the cell surface [16].

Once ligand-bound dimeric complexes are formed, their activation leads to the initiation of a signalling pathway. From a biological perspective, this activation is usually the result of a sequence of phosphorylation events, involving different tyrosine residues on the intra-cellular tails of the receptors forming the dimer. From a mathematical perspective, this sequence of events is usually neglected by considering instantaneous phosphorylation [5,13]. This is described in §2.1, where the IP model is described. However, we also consider an extension of this model in §2.2, the DP model, where the phosphorylation of ligand-bound dimeric complexes is considered as an additional reaction. We refer the reader to MacGabhann & Popel [15] for a brief discussion on the importance of including phosphorylation, and to Bel *et al*. [17] for a discussion of the conditions under which the sequence of phosphorylation events can be treated as a single reaction.

For the IP and DP models, the aim in §2.1 and 2.2, as well as appendices B and C, is to compute the time to reach a given signalling threshold, where the amount of signalling in the process is identified with the number of phosphorylated (either instantaneously or not) complexes at any given time. Moreover, the steady-state distribution of the system is also computed. Finally, a sensitivity analysis of both models is carried out in appendix D, to quantify how the association, dissociation, phosphorylation and de-phosphorylation rates affect the dynamics of the receptor–ligand system.

The study of the number of ligand-bound monomeric, non-phosphorylated and phosphorylated ligand-bound dimeric molecules on the cell surface over time can be viewed as the analysis of the transient behaviour of a specific multi-variate Markov process, a problem which, in general, is not solvable in closed form [18]. Therefore, one typically carries out Gillespie simulations [19], or applies moment-closure techniques [20,21] to deal with the master equation of the Markov process under study. In this study, and for the models considered in §2.1 and 2.2, we apply alternative methods, which allow us to analyse, in an exact way, the quantities of interest mentioned above. In particular, by considering the time to reach a given signalling threshold as a continuous random variable, and by conveniently structuring the space of states of the continuous time Markov processes under study, we identify this time as the absorption time in an auxiliary absorbing continuous time Markov process. We compute the Laplace–Stieltjes transforms of this random variable, as well as the steady-state probabilities, by making use of first-step and matrix-analytic arguments. A novel local sensitivity analysis for the Markov processes considered is adapted and applied here by generalizing arguments from Caswell [22] (see also [23]). This analysis allows us to quantify how the stochastic descriptors considered in §3.3, time to signalling threshold and steady-state probability distribution, are affected by the association, dissociation, phosphorylation and de-phosphorylation rates.

### 2.1. IP model: instantaneous phosphorylation

**Figure 1.**
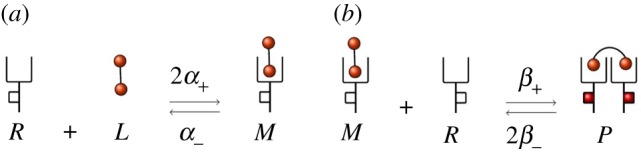
Reactions of the IP model. (*a*) Association and dissociation of bound monomers (*M*). (*b*) Association and dissociation of bound dimers (*P*), which instantaneously phosphorylate (represented by red squares as phosphorylated residues in the intra-cellular tail of the receptors).

In this section, we consider a model of a bivalent ligand that can bind a free receptor to form a bound monomer (or *M* complex). Receptors can diffuse on the cell surface, so that eventually a free receptor can bind an extracellular ligand to form a bound monomer *M*. This complex in turn can further engage a second receptor to form a ligand-bound and cross-linked receptor dimer (or *P* complex). Once a *P* complex is formed, it is instantaneously phosphorylated, so that *P* complexes on the plasma membrane initiate signalling, in the spirit of Starbuck *et al.* [2] and Alarcón & Page [5]. Ligand-bound monomers and dimers can dissociate. We assume that de-phosphorylation of *P* takes place when cross-linked receptor dimers also dissociate. In this scenario, four possible reactions can occur with different association and dissociation rates as shown in figure 1.

In what follows, we consider an environment with constant number, *n*_R_ and *n*_L_, of receptors and ligands, spatially well-mixed on the cell surface and in the extracellular space, respectively. We are interested in the number of *M* and *P* complexes on the cell surface as a function of time, which we model using a stochastic approach: as a continuous time Markov chain (CTMC) 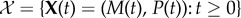, where *M*(*t*) and *P*(*t*) represent the number of *M* and *P* complexes, respectively, at time *t*. We note that, if we define the random variables *R*(*t*) and *L*(*t*) as the numbers of free receptors and ligands, respectively, at time *t* ≥ 0, it is clear that *R*(*t*) = *n*_R_ − *M*(*t*) − 2*P*(*t*) and *L*(*t*) = *n*_L_ − *M*(*t*) − *P*(*t*), for all *t* ≥ 0. Then, *R*(*t*) and *L*(*t*) are implicitly analysed in 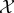 and do not need to be explicitly considered in the CTMC. We need to impose the conditions *M*(*t*), *P*(*t*) ≥ 0 and, from the previous comments, we have
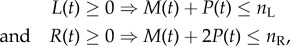
for all *t* ≥ 0, which specify the state space 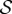 of 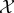. Specifically, we note that given (*M*(*t*), *P*(*t*)) = (*n*_1_, *n*_2_) at some time *t* ≥ 0, then

— if 2*n*_L_ ≤ *n*_R_: *n*_1_ + *n*_2_ ≤ *n*_L_ ⇒ *n*_1_ + 2*n*_2_ ≤ *n*_R_ and
— if *n*_R_ ≤ *n*_L_: *n*_1_ + 2*n*_2_ ≤ *n*_R_ ⇒ *n*_1_ + *n*_2_ ≤ *n*_L_,

so that three different specifications of the state space 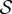 are obtained, depending on the particular values of *n*_R_ and *n*_L_. In particular:

— if 2*n*_L_ ≤ *n*_R_, then 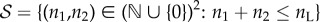,
— if *n*_R_ < 2*n*_L_ < 2*n*_R_, then 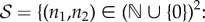 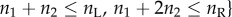 and
— if *n*_R_ ≤ *n*_L_, then 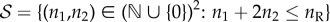.

**Figure 2.**
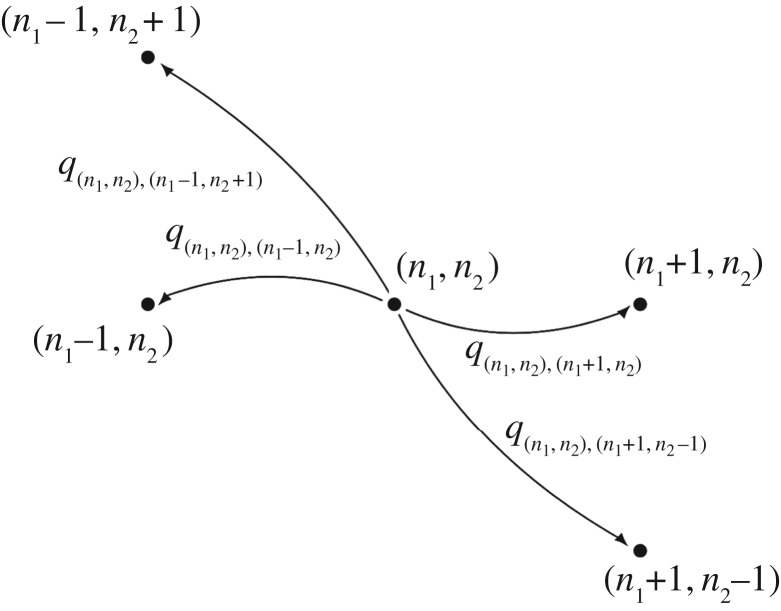
Transition diagram for the IP model (process 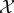).

Although we can deal with each of these cases, without loss of generality, we focus here on the first one, 2*n*_L_ ≤ *n*_R_, since these are the physiological conditions for the receptor–ligand system analysed in §3. Thus, the stochastic process 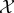 is defined over 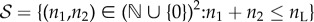. From figure 1, it is clear that transitions from states in the interior of 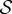, that is, from states 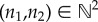 with *n*_1_ + *n*_2_ < *n*_L_, can take place to four adjacent states as shown in figure 2. Transitions for states within the boundary of 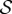 are obtained in a similar way by discarding those transitions that leave 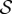.

Transitions between states in our CTMC are governed by the infinitesimal transition rates *q*_(*n*_1_,*n*_2_),(*n*_1_′,*n*_2_′)_, with 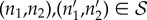. These infinitesimal transition rates are obtained by mass action kinetics, and by the fact that if the process is in state (*n*_1_, *n*_2_) at a given time, there are (*n*_L_ − *n*_1_ − *n*_2_) free ligands and (*n*_R_ − *n*_1_ − 2*n*_2_) free receptors available. The formation of *M* complexes depends on the number of free receptors and ligands, and their dissociation only depends on the number of *M* complexes. A similar analysis can be made for *P* complexes. Finally, we note that the formation of *M* complexes and dissociation of *P* complexes can take place with any of the two available binding sites of the ligand. Then, the specific values of the non-null infinitesimal transition rates are given by2.1
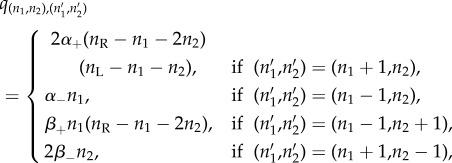
where *α*_+_, *α*_−_, *β*_+_ and *β*_−_ are positive constants representing the association and dissociation rates for *M* and *P* complexes, respectively.

For this model, the focus in §3.3 is on several summary statistics (or stochastic descriptors) that allow one to study the timescales for signal initiation on the cell membrane, as well as the late time behaviour of the system, and to carry out a local sensitivity analysis to test how these summary statistics depend on the different parameters (e.g. kinetic rates) of the model. An efficient matrix-oriented analysis of these summary statistics, for the IP model, can be found in appendix B.

### 2.2. DP model: delayed phosphorylation

**Figure 3.**
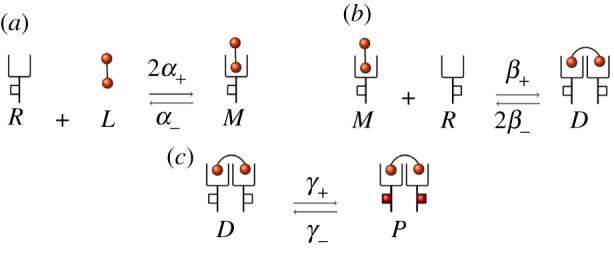
Reactions of the DP model. (*a*) Association and dissociation of bound monomers (*M*). (*b*) Association and dissociation of non-phosphorylated bound dimers (*D*). (*c*) Phosphorylation and de-phosphorylation of phosphorylated bound dimers (*P*).

In the previous section, the *P* complexes were instantaneously phosphorylated. Here we relax this requirement and include phosphorylation as an additional reaction (figure 3). We note that, in the DP model presented in figure 3, dissociation of phosphorylated receptors can only occur after their de-phosphorylation. One may alternatively consider that dissociation can occur due to ligand unbinding to one of the receptors, even if de-phosphorylation has not occurred yet. For this case, a similar analysis to the one carried out in this section could be developed, and bound phosphorylated monomers should be incorporated as a new molecular *species*. Numerical results for the VEGFR2 receptor and VEGF-A ligand system (§3), including this additional molecular species and not reported here, show similar qualitative dynamics to the simpler model considered in this section.

In what follows, we adapt the arguments of the previous section to the DP model. This not only allows us to evaluate the relevance of phosphorylation as an independent reaction (with numerical results presented in §3), but also serves as an example of how to include new reactions in this type of stochastic model, while adapting the matrix-analytic arguments.

In brief, we consider the CTMC 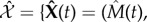 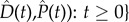, where
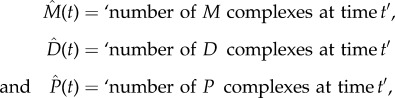
for all *t* ≥ 0, where *D* complexes refer to non-phosphorylated bound dimers and *P* to phosphorylated ones. From the reactions in figure 3, it is clear that for all *t* ≥ 0
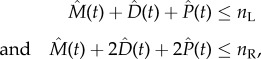
and, by assuming as previously, that 2*n*_L_ ≤ *n*_R_, it is easy to show that
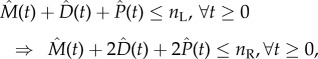
so that 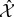 is defined over 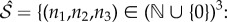 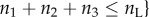.

**Figure 4.**
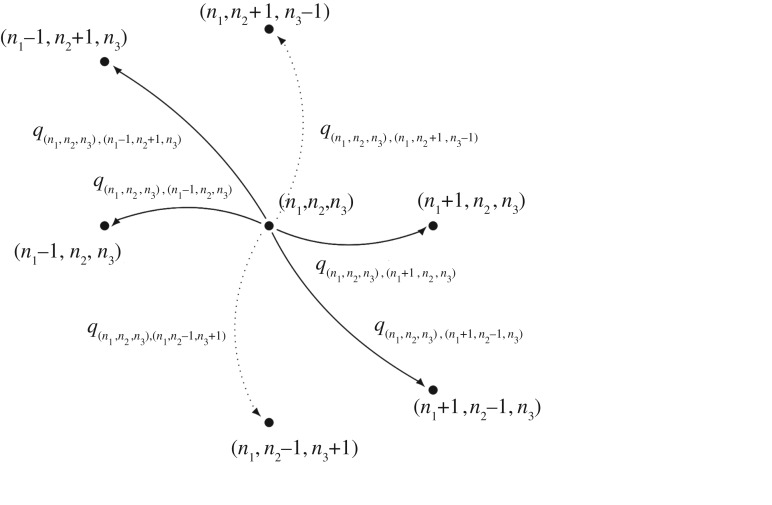
Transition diagram for the DP model (process 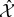).

From figure 3, the transition diagram can be obtained (figure 4), where the non-null infinitesimal transition rates are obtained in a manner analogously to (2.1). In particular, we have

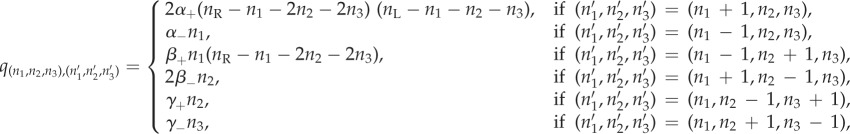


where *α*_+_, *α*_−_, *β*_+_, *β*_−_, *γ*_+_ and *γ*_−_ are positive constants representing the association, dissociation and phosphorylation rates for the complexes in figure 3. Similar summary statistics to those studied for the IP model, and analysed in §3.3, are analysed for the DP model in appendix C, by following a matrix-oriented approach.

## 3. The vascular endothelial growth factor receptor–ligand system

In this section, we illustrate the analytical work developed in the previous ones and the appendices, and focus on the interaction between VEGFR2 receptors and VEGF-A ligands on the surface of human umbilical vein endothelial cells (HUVECs), an interaction initiating signalling cascades that can cause diverse cellular responses, such as cell motility, division or death (i.e. apoptosis). We first develop, in §3.1, a method to estimate the parameters *α*_+_, *α*_−_, *β*_+_ and *β*_−_ for the interaction between the VEGFR2 receptor and the VEGF-A ligand molecule. We do so by making use of the methods proposed by Lauffenburger and Linderman [1], where the transport mechanism of free ligand or free receptor is modelled by molecular diffusion, and where diffusive transport dominates convective transport caused by fluid motion at cellular and sub-cellular length scales [1,24].

The rates estimated in §3.1 depend on several physiological parameters, which are presented in §3.2. In §3.3, we analyse a number of stochastic descriptors of interest when the IP or the DP models are considered for this interaction. This allows us to study the impact of phosphorylation as a separate reaction in the process (delayed phosphorylation), to quantify timescales for signalling initiation under different ligand concentrations and to analyse the impact that each kinetic rate has in these stochastic descriptors. Finally, we investigate in §3.4 the effect that synthesis of new free receptors on the cell surface, and internalization of bound complexes into endosomal compartments, can have on the molecular dynamics.

### 3.1. Estimation of association and dissociation rates

**Figure 5.**
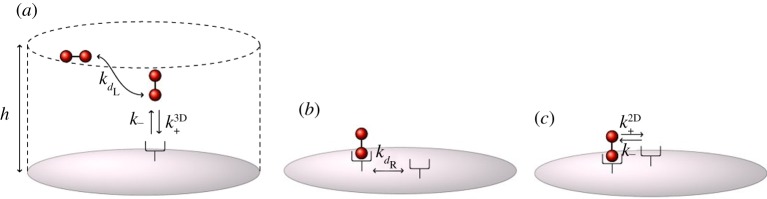
(*a*) Two-step binding and unbinding of receptor and ligand: *k*_*d*_L__ is the ligand transport rate, *k*^3D^_+_ and *k*_−_ are the intrinsic binding and unbinding rates, respectively, and *h* is the characteristic length of the experimental volume. (*b*) Diffusive transport of surface receptor: *k*_*d*_R__ is the transport rate for both receptor *R* and bound monomer *M*. (*c*) Once in the reaction zone of *M*, *R* can bind with rate *k*^2D^_+_ (which is a *2D version* of *k*^3D^_+_) or unbind with rate *k*_−_.

We estimate in §3.3 the parameters *α*_+_, *α*_−_, *β*_+_ and *β*_−_ (*s*^−1^) for the binding and unbinding of the VEGFR2 receptor and its bivalent VEGF-A ligand. We consider a fraction, 0 < *f* < 1, of a HUVEC, for computational reasons, and denote the receptor molecule by *R* and the ligand by *L*. Firstly, we set the dissociation rate *k*_off_ = 1.32 × 10^−3^ s^−1^ as reported in MacGabhann & Popel [15] for VEGFR2. From the equilibrium dissociation rate, *K*_*d*_ (mm^−3^ mol), given by *K*_*d*_ = *k*_off_/*k*_on_, it is possible then to obtain the biophysical binding rate, *k*_on_ (mol^−1^ mm^3^ s^−1^). Therefore, the transition rates *α*_+_ and *α*_−_ of §2 are given by
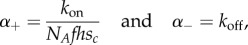
where *h* (mm) is the characteristic length of the experimental volume, *s*_*c*_ (mm^2^) is the total area of the cell surface and *N*_A_ (mol^−1^) is Avogadro's number. In order to estimate the transition rates *β*_+_ and *β*_−_, we first note that the binding process between the receptor and the ligand, such as reaction (*a*) in figure 1, can be considered as a one-step process, with *q*_on_ (mm^3^ s^−1^) the association constant and *q*_off_ (s^−1^) the dissociation constant. Constants *q*_on_ and *q*_off_ are related to the biophysical rates *k*_on_ and *k*_off_ as follows:
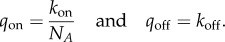
However, these binding and unbinding events are in fact two-step processes [1,25–29]. In the first step, the ligand and the receptor simply encounter each other; that is, ligands diffuse into a sufficiently close proximity of the receptor to allow the chemical reaction step to occur. Let us define the ligand diffusion rate *k*_*d*_L__ (mm^2^ s^−1^), and the 3D reaction intrinsic rate *k*^3D^_+_ (mm^3^ s^−1^). The mechanism of the reverse process is similar, so that the unbinding of the receptor and the ligand occurs with intrinsic dissociation rate *k*_−_ (s^−1^) and the outward diffusion with transport rate *k*_*d*_L__ (figure 5*a*).

As mentioned earlier, we restrict our study to a fraction 0 < *f* < 1 of the cell surface, so that the radius of this target surface is given by
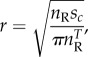
where *n*^*T*^_R_ is the total number of receptors on the cell surface, and *n*_R_ = *fn*^*T*^_R_ is the number of receptors present on the target surface. We have assumed, thus, an homogeneous spatial distribution of VEGFR2 on the cell surface [30,31], neglecting receptor clustering, which might be initiated upon ligand stimulation [32]. Under this assumption, the contributions of rates *k*_*d*_L__, *k*^3D^_+_ and *k*_−_ to the overall association and dissociation rates, *q*_on_ and *q*_off_, respectively, are given by3.1
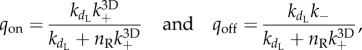
where *k*_*d*_L__ = 4*πD*_L_*r*, as shown elsewhere [1,25–28]. We note that *q*_on_ is a *per receptor* rate, as explained elsewhere [1,33]. A similar argument (figure 5*b*, *c*) applies when computing the rate of free receptor binding (*k*_c_ (mm^2^ s^−1^)) or unbinding (*k*_u_ (s^−1^)) to a monomer on the cell membrane [1], which occurs with rates3.2
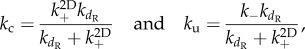
where the transport rate *k*_*d*_R__ (mm^2^ s^−1^) (figure 5*b*) is given by *k*_*d*_R__ = 2*πD*/log(*w*/*b*). The diffusion constant *D* = *D*_R_ + *D*_M_ (mm^2^ s^−1^) is the sum of the diffusivities of the receptor and the bound monomer on the cell membrane (which are assumed to be the same *D*_R_ = *D*_M_), *b* (mm) is the characteristic length of the receptor, and *w* (mm) is one-half the mean distance between receptors, given by
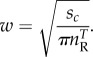
We find *k*^3D^_+_ and *k*_−_ from equation (3.1). Once *k*^3D^_+_ is in hand, the intrinsic 3D binding rate allows to compute its 2D version, *k*^2D^_+_, as follows:
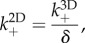
where *δ* (mm) is the cell membrane thickness, as suggested in Lauffenburger & Linderman [1]. Given *k*^2D^_+_, rate constants *k*_c_ and *k*_u_ can be found by means of equation (3.2). Finally, these rates, *k*_c_ and *k*_u_, are related to *β*_+_ and *β*_−_, respectively, for the CTMCs considered in §2, as follows:
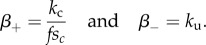


### 3.2. Physiological parameters

All the rates of the IP and DP models (figures 1 and 3, respectively) used in §3.3 and §3.4 have been obtained following the approach described in §3.1, with physiological parameters taken from the literature. In particular, physiological parameters are given in table 1, and the specific rates for the IP and DP models are given in table 2. The equilibrium dissociation rate for VEGF-A and VEGFR2 is equal to *K*_*d*_ = 150 pM, as reported in MacGabhann & Popel [15]. This rate is consistent with previously reported values for *in silico* experiments [37], and agrees with experimentally determined ones [38–41].

**Table 1.** Physiological parameters.

physiological parameter	value	reference
endothelial cell surface area, *s*_c_	10^−3^ mm^2^	[15]
VEGF-A diffusion coefficient at 4°C, *D*_L_	5.2 × 10^−5^ mm^2^ s^−1^	[34]
VEGFR2 diffusion coefficient, *D*_R_	10^−8^ mm^2^ s^−1^	[35]
VEGFR2 radius, *b*	5 × 10^−7^ mm	[5]
average membrane thickness of ECs, *δ*	10^−4^ mm	[36]
characteristic length of the experimental volume, *h*	1 mm	[15]
dissociation rate, *k*_off_	1.32 × 10^−3^ s^−1^	[15]
equilibrium dissociation rate, *K*_d_ for VEGFR2	1.5 × 10^−16^ mm^−3^ mol	[15]
phosphorylation rate for *D* complexes, *γ*_+_	3.67 × 10^−3^ s^−1^	[1]
de-phosphorylation rate for *P* complexes, *γ*_−_	9.17 × 10^−4^ s^−1^	[1]

**Table 2.** Rates (in s^−1^) for the IP and DP models, considering *f* = 4% of the cell surface. Note that parameters *γ*_+_ and *γ*_−_ are not considered in the IP model.

reactions of the IP model	*α*_+_	3.653 × 10^−7^
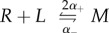	*α*_−_	1.320 × 10^−3^
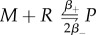	*β*_+_	4.483 × 10^−4^
reactions of the DP model	*β*_−_	1.620 × 10^−4^
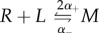	*γ*_+_	3.667 × 10^−3^
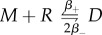	*γ*_−_	9.167 × 10^−4^
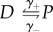		

We consider in this section the subset of endothelial cells, called human umbilical vein endothelial cells (HUVECs), which have been characterized to express (on average) 5800 VEGFR2s per cell [42]. We focus on 4% of the cell surface (*f* = 0.04) for computational reasons, so that in this area the total number of VEGFR2s is *n*_R_ = 232. For the IP and DP models, our numerical results should be considered exact, since they have been obtained making use of the analytical arguments described in the appendices.

### 3.3. Results

In this section (both for the IP and the DP models), we focus on two stochastic descriptors (or summary statistics) that allow one to study the timescales for signal initiation on the cell surface (in terms of phosphorylated dimers), as well as the late time behaviour of the system (in terms of the steady-state number of free receptors, monomers and dimers). In particular, we focus on

(1) Starting in any state **n** (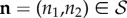 for the IP model, and 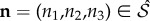 for the DP model), the time *T*_**n**_(*N*) to reach, for the first time, *N* phosphorylated dimers on the cell surface; that is, *T*_**n**_(*N*) = inf{*t* ≥ 0 : *P*(*t*) = *N*} for the IP model, and 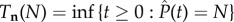 for the DP model.
(2) The stationary probability distribution of the system, which does not depend on the initial conditions; that is, the probabilities 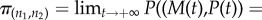 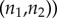 for the IP model and 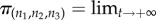 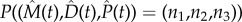 for the DP model.

We note that in this section we always consider initial states such that (*n*_1_, *n*_2_) = (0, 0) and (*n*_1_, *n*_2_, *n*_3_) = (0, 0, 0). These initial conditions indicate that at time *t* = 0 (when ligand stimulation occurs), all receptors are in monomeric form. We report in appendices B and C a matrix-oriented approach to study these summary statistics for the IP and the DP model, respectively, and in appendix D, a matrix-oriented method to carry out a local sensitivity analysis of these summary statistics with respect to the model parameters (e.g. kinetic rates). This allows one to explore what the contribution is of each kinetic rate to a given stochastic descriptor.

#### 3.3.1. Time to reach a signalling threshold

**Figure 6.**
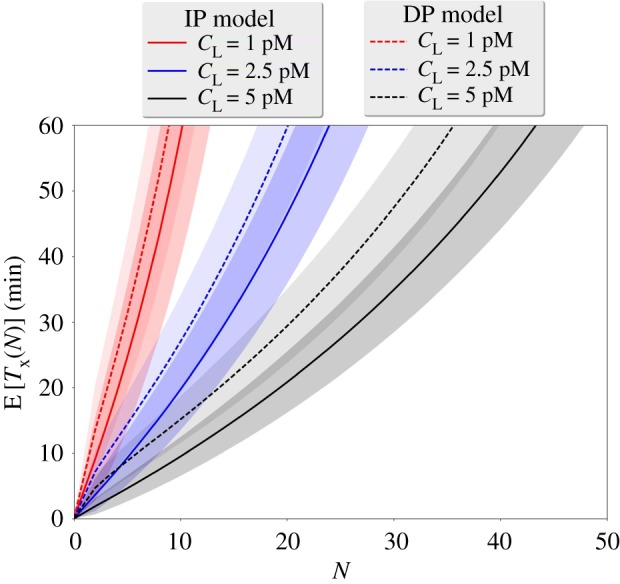
*E*[*T*_**x**_(*N*)] for (from *left* to *right*) ligand concentrations, *c*_L_ ∈ {1 pM, 2.5 pM, 5 pM}, and for the IP model (*solid* curves) and the DP model (*dashed* curves). The initial state for the IP model is **x** = (0, 0) and for the DP model it is **x** = (0, 0, 0).

In figure 6, we plot *E*[*T*_(0,0)_(*N*)] (for the IP model) and *E*[*T*_(0,0,0)_(*N*)] (for the DP model), for values 0 ≤ *N* ≤ *n*_L_, where *n*_L_ ∈ {23, 58, 116} is the number of ligands considered, which corresponds to the following ligand concentrations, *c*_L_ ∈ {1 pM, 2.5 pM, 5 pM}. We note that these concentrations are similar to those reported in serum for healthy controls and cancer studies (see table I in [43]). The three different values of *n*_L_ correspond to 10%, 25% and 50% of *n*_R_, the total number of VEGFR2 on the fraction of the cell surface considered. The number of ligands, thus, verifies the condition 2*n*_L_ ≤ *n*_R_, assumed in the analysis of *T*_(0,0)_(*N*), as discussed in §2.1. *T*_(0,0)_(*N*) is the continuous random variable that represents the time to reach a total number, *N*, of phosphorylated bound dimers, *P*, given the initial state (0, 0), in the IP model where instantaneous phosphorylation is considered (for details, see §2.1), while *T*_(0,0,0)_(*N*) is its DP model counterpart. Our results have been restricted to times up to 60 min, to describe the early time dynamics on the cell surface. The late time behaviour of the system will be analysed by means of its steady-state distribution. In figure 6, *solid* curves represent the values of *E*[*T*_(0,0)_(*N*)], while dashed curves represent the values of *E*[*T*_(0,0,0)_(*N*)], obtained by means of algorithm 1 (see appendix B). Shaded areas have been obtained for both models by considering *E*[*T*_**x**_(*N*)] ± *SD*[*T*_**x**_(*N*)], where *SD*[*X*] represents the standard deviation of the random variable *X*, also obtained from algorithm 1.

In figure 6, a monotonic behaviour is observed. For a fixed value of *N* in the IP model, *E*[*T*_(0,0)_(*N*)] is always smaller for larger ligand concentrations, *c*_L_. Indeed, an increase in the amount of available ligand to bind receptors implies reaching the given signalling threshold (encoded by *N*) in a shorter time. The behaviour for *E*[*T*_(0,0,0)_(*N*)] is similar to that observed for *E*[*T*_(0,0)_(*N*)], so that the consideration of delayed phosphorylation in the DP model does not seem to qualitatively affect the main features of this descriptor. This can be explained as follows: the most likely fate of a bound monomer is to phosphorylate before its dissociation. However, the consideration of phosphorylation as an additional reaction delays the time to reach a given threshold *N* and every curve for the DP model is displaced to the left of its corresponding one for the IP model. For example, for *c*_L_ = 1 pM, the mean time *E*[*T*_(0,0)_(*N*)] to reach a threshold *N* = 5 (20% of *n*_L_) of phosphorylated bound dimers is approximately 25 min under the IP model. When the phosphorylation of bound complexes is explicitly considered (DP model), this mean time increases approximately up to 31 min.

#### 3.3.2. Stationary probability distribution

The asymptotic behaviour of the curves shown in figure 6 is directly related to the maximum signalling threshold that is, in fact, reached by the process in short and intermediate timescales. From a purely mathematical perspective, any state within 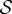 (or 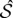 in the DP model) is reached in the IP model (DP model) as 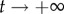, since 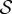 (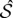) is an irreducible finite class of states for the process 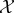 (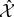). However, according to our numerical results, there exists a subset of (high) signalling thresholds that is not reached in practice by 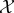 (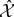). This maximum signalling threshold is encoded in the steady-state probability distribution of this process, which can be computed from algorithm 2 (see appendix B), and which measures the potential of the system to reach any signalling threshold at sufficiently late times, for different ligand concentrations.

**Figure 7.**
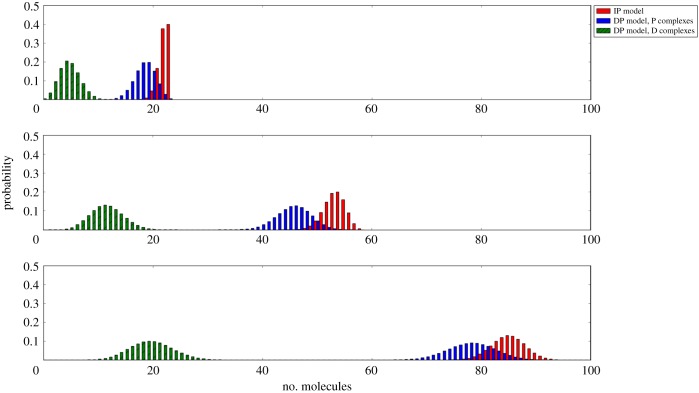
Probability distribution of the number of bound dimers in steady state for the processes 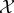 (IP model, P bound dimers, *red*) and 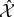 (DP model, D and P bound dimers, *green* and *blue*) for (from *top* to *bottom*) ligand concentrations, *c*_L_ ∈ {1 pM, 2.5 pM, 5 pM}.

In figure 7, the distribution of the number of (phosphorylated and non-phosphorylated) bound dimers at steady state, for the IP and the DP models, is plotted for different ligand concentrations, *c*_L_ ∈ {1 pM, 2.5 pM, 5 pM}. For low ligand concentrations, nearly all the *n*_L_ available ligands are forming phosphorylated bound dimers in steady state. This is particularly the case in the IP model, where no non-phosphorylated bound dimers exist. In the DP model, a small number of non-phosphorylated bound dimers can be found in steady state. These non-phosphorylated bound dimers in steady state explain why the distribution of the number of phosphorylated bound dimers in steady state is displaced to the left when phosphorylation is considered as a separate reaction in the DP model, in comparison with the same distribution in the IP model.

#### 3.3.3. Dynamics of the receptor–ligand system

**Figure 8.**
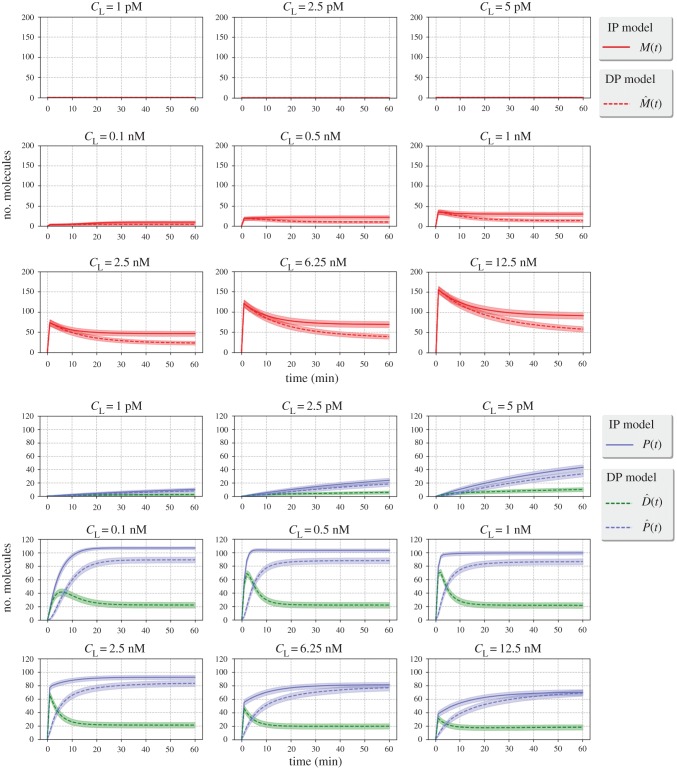
Gillespie simulations of the processes 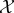 and 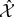 for different initial ligand concentrations, *c*_L_ ∈ {1 pM, 2.5 pM, 5 pM, 0.1 nM, 0.5 nM, 1 nM, 2.5 nM, 6.25 nM, 12.5 nM}. *Dashed* lines correspond to the IP model and *solid* lines correspond to the DP model. Time course for bound monomers (*top*) and dimers (*bottom*).

We now complement our previous results by carrying out a number of Gillespie simulations of the models, so that the time course of the different random variables in our processes (*M*(*t*), *P*(*t*), 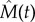, 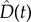 and 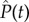) can be studied. In particular, we plot in figure 8 the mean plus and minus (shadowed area) the standard deviation of the variables of interest (*M*(*t*) and *D*(*t*) in the IP model, and 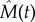, 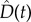 and 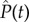 in the DP model). The time course has been generated by means of Gillespie simulations, where we have broadened the VEGF-A concentration range by considering *n*_L_ ∈ {0.1*n*_R_, 0.25*n*_R_, 0.5*n*_R_, 10*n*_R_, 50*n*_R_, 100*n*_R_, 250*n*_R_, 625*n*_R_, 1250*n*_R_}, which approximately corresponds to concentrations *c*_L_ ∈ {1 pM, 2.5 pM, 5 pM, 0.1 nM, 0.5 nM, 1 nM, 2.5 nM, 6.25 nM, 12.5 nM}. We note that for small ligand concentrations the number of bound dimers grows as the VEGF-A concentration increases. For concentrations *c*_L_ ∈ {1 pM, 2.5 pM, 5 pM} the steady state is not reached in the first 60 min of the numerical simulations (figures 7 and 8). However, higher concentrations result in a saturated scenario, where we obtain lower numbers of *P* complexes for ligand concentrations higher than *c*_L_∼2.5 nM. Thus, concentrations around 0.1 nM − 2.5 nM may be considered as optimum when only surface dynamics of phosphorylated bound dimers is of interest.

As mentioned above, for ligand concentrations around *c*_L_ ∈ {6.25 nM, 12.5 nM}, the system exhibits a reduction in the number of bound dimers, which is caused by the fast and early formation of monomeric bound complexes (figure 8). In fact, for both IP and DP models and when focusing on the formation of bound monomers as a function of time, we observe, under optimum ligand concentrations, a peak of monomeric complexes in the first 5 min, which is followed by a decrease to the steady-state values. The same early peak can be observed under these ligand concentrations for non-phosphorylated bound dimers in the DP model, which is followed by an increase in the number of phosphorylated bound dimers. For high ligand concentrations, the steady-state value for monomeric complexes increases, so that formation of bound dimers is effectively blocked. The inhibition of bound dimer formation at high ligand concentrations is intrinsically related to the ligand-induced-dimerization assumption, where the formation of free receptor pre-dimers is not allowed. However, if free receptor pre-dimers were to be considered, their effect would be negligible for ligand concentrations below 1 nM [15], as our results in figure 8 also suggest.

#### 3.3.4. Local sensitivity analysis

We study in this section the effect of the association, dissociation, phosphorylation and de-phosphorylation rates on the descriptors introduced, which can be estimated by means of the sensitivity analysis proposed in appendix D. In table 3, we present the elasticities (i.e. normalized derivatives) of the descriptors *E*[*T*_(0,0)_(*N*)], *E*[*T*_(0,0,0)_(*N*)], *π*_*P*_ and 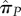 (see appendices B and C), when *N* is chosen to be 25% of the total number of ligands *n*_L_, and for different ligand concentrations, *c*_L_. As expected, the effect of each rate on any descriptor increases with increasing values of ligand concentration. It is also worth noting that the elasticities of the mean number of phosphorylated complexes in steady state are equal, with opposite sign, with respect to the association and dissociation rates (e.g. (∂*π*_*P*_/∂*α*_+_)/(*π*_*P*_/*α*_+_) = −(∂*π*_*P*_/∂*α*_−_)/(*π*_*P*_/*α*_−_)), which means that this variable only depends on the ratio of parameters: *α*_+_/*α*_−_, *β*_+_/*β*_−_ and *γ*_+_/*γ*_−_. This can be easily understood since, from a deterministic perspective, the steady state corresponding to the DP model can be obtained as the solution of the following system of equations:
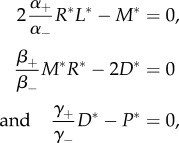
which only depends on these parameter ratios. We also note that, according to the results of table 3, the rate *α*_+_ plays an important role in all the descriptors. This can be explained as follows: once a ligand is ‘destined’ to form a bound monomer complex, its most likely fate is to lead to a phosphorylation event before dissociation of the corresponding dimer occurs (see discussion in §4).

**Table 3.** Elasticities for the stochastic descriptors *E*[*T*_(0,0)_(*N*)] and *E*[*T*_(0,0,0)_(*N*)] and mean values *π*_P_ and 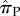, with respect to each parameter, *θ*_*i*_ ∈ {*α*_+_, *α*_−_, *β*_+_, *β*_−_, *γ*_+_, *γ*_−_} for different ligand concentrations, *c*_L_ ∈ {1 pM, 2.5 pM, 5 pM}.

elasticity	*c*_L_	*α*_+_	*α*_−_	*β*_+_	*β*_−_	*γ*_+_	*γ*_−_
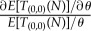	1 pM	−9.98 × 10^−1^	1.61 × 10^−2^	−2.17 × 10^−2^	3.42 × 10^−3^	—	—
	2.5 pM	−9.99 × 10^−1^	1.78 × 10^−2^	−2.36 × 10^−2^	4.60 × 10^−3^	—	—
	5 pM	−1.00	2.01 × 10^−2^	−2.66 × 10^−2^	6.02 × 10^−3^	—	—
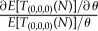	1 pM	−8.47 × 10^−1^	1.22 × 10^−2^	−1.73 × 10^−2^	2.12 × 10^−3^	−2.26 × 10^−1^	8.82 × 10^−2^
	2.5 pM	−8.60 × 10^−1^	1.33 × 10^−2^	−1.84 × 10^−2^	2.59 × 10^−3^	−2.68 × 10^−1^	1.36 × 10^−1^
	5 pM	−8.72 × 10^−1^	1.51 × 10^−2^	−2.07 × 10^−2^	3.30 × 10^−3^	−2.99 × 10^−1^	1.76 × 10^−1^
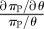	1 pM	3.45 × 10^−2^	−3.45 × 10^−2^	3.82 × 10^−2^	−3.82 × 10^−2^	—	—
	2.5 pM	6.67 × 10^−2^	−6.67 × 10^−2^	7.17 × 10^−2^	−7.17 × 10^−2^	—	—
	5 pM	1.03 × 10^−1^	−1.03 × 10^−1^	1.10 × 10^−1^	−1.10 × 10^−1^	—	—
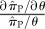	1 pM	7.31 × 10^−3^	−7.31 × 10^−3^	8.08 × 10^−3^	−8.08 × 10^−3^	2.06 × 10^−1^	−2.06 × 10^−1^
	2.5 pM	1.73 × 10^−2^	−1.73 × 10^−2^	1.85 × 10^−2^	−1.85 × 10^−2^	2.15 × 10^−1^	−2.15 × 10^−1^
	5 pM	5.88 × 10^−2^	−5.88 × 10^−2^	6.12 × 10^−2^	−6.12 × 10^−2^	2.49 × 10^−1^	−2.49 × 10^−1^

### 3.4. A study of receptor internalization and synthesis

**Figure 9.**
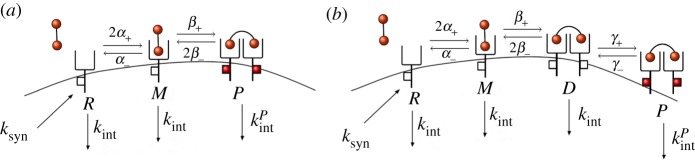
IP and DP models when synthesis of free receptors, as well as internalization of free receptors, monomers and dimers can take place. (*a*) The extended IP model and (*b*) the extended DP model.

It is well known that rapid internalization occurs for VEGFR2 following ligand binding and phosphorylation [39]. We briefly explore in this section how receptor synthesis and internalization events can have an impact on the molecular dynamics of the cell surface. In figure 9, we represent the IP and the DP models under the assumption that synthesis of new receptors, as well as internalization of free receptors, monomers and dimers, can also take place. We note that since modelling endosomal compartments is out of the scope of this paper, recycling events have not been explicitly considered in what follows: this would require tracking down the number of molecules in the different intra-cellular compartments, and thus, additional variables in the stochastic models. However, one can interpret the synthesis rate *k*_*syn*_ in figure 9 as an *insertion rate* [15], which implies a net contribution of new receptors on the cell surface, without having to specify whether these receptors have been truly synthesized and transported to the surface from the Golgi apparatus, or have been recycled to the surface from endosomal compartments. Since the parameter *n*_R_ is the basal (i.e. under no ligand stimulation) number of receptors on the cell surface, internalization and synthesis rates need to satisfy the condition *k*_syn_ = *n*_R_*k*_int_. Moreover, we set *k*_int_ = 2.8 × 10^−4^ s^−1^ as previously determined [44], and consider that phosphorylated dimers can be internalized faster than non-phosphorylated ones [15,45], by setting *k*^P^_int_ = *qk*_int_ with *q* ∈ {1.0, 2.0, 5.0, 10.0} (figure 9).

**Figure 10.**
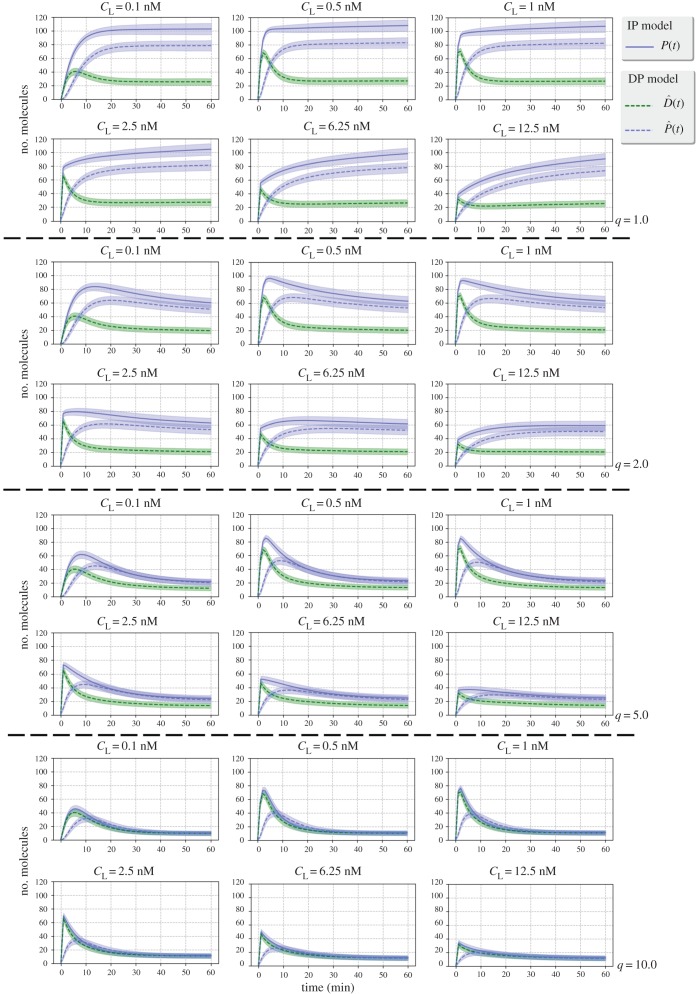
Gillespie simulations for the extended IP and DP models of figure 9, for different initial ligand concentrations, *c*_L_ ∈ {0.1 nM, 0.5 nM, 1 nM, 2.5 nM, 6.25 nM, 12.5 nM} and different values of *q* ∈ {1, 2, 5, 10}. *Dashed* lines correspond to the IP model and *solid* lines correspond to the DP model. Time course for phosphorylated and non-phosphorylated dimers.

In figure 10, we plot analogous results to those of figure 8 for the models considered in figure 9 and values *q* ∈ {1, 2, 5, 10}. We focus here on the dynamics of phosphorylated (*P*(*t*) in the IP model, 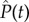 in the DP model) and non-phosphorylated (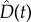 in the DP model) dimers, and consider concentrations *c*_L_ ∈ {0.1 nM, 0.5 nM, 1 nM, 2.5 nM, 6.25 nM, 12.5 nM}. If internalization of phosphorylated dimers does not occur fast enough (e.g. values *q* ∈ {1.0, 2.0} in figure 10), a steady-state pool of phosphorylated dimers is maintained at late times on the cell surface. Under faster internalization (*q* ∈ {5.0, 10.0}), and for optimum ligand concentrations, a peak of phosphorylated dimers is observed after ligand stimulation (at time 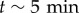 for the IP model and at time 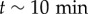 for the DP model). It is interesting to observe that the peak of non-phosphorylated dimers is well captured in figure 8 (i.e. when internalization and synthesis are not considered), and the same is true for the time course of monomers (not reported in figure 10). It is only the peak of phosphorylated dimers which is significantly affected by internalization dynamics. Equally, optimum ligand concentrations are well characterized by the original IP and DP models; that is, similar optimum ligand concentrations are found, of the order of approximately 1 nM, in figures 8 and 10 (i.e. with and without receptor synthesis and internalization).

## 4. Discussion

In this paper, our aim was to quantify the signalling timescales (or phosphorylation) for two different stochastic models of receptor–ligand interaction (instantaneous phosphorylation, IP model, and delayed phosphorylation, DP model), and to analyse their late time behaviour, making use of new exact matrix-analytic techniques. Stochastic approaches are essential in order to explore the role of limited (and small) protein copy numbers in receptor–ligand signalling systems, since the stochastic nature of protein expression and quantitative differences in the abundance of proteins could dysregulate receptor-mediated signalling, as recently reported by Shi *et al.* [46].

We have assumed that bound dimers are instantaneously phosphorylated in the IP model, while in the DP model phosphorylation is considered a new and independent reaction. In these two models, matrix-analytic techniques have been applied (see appendices B and C, respectively) to study the time to reach a threshold number of phosphorylated bound dimers, *P*, on the cell membrane, and the steady-state probability distribution. We have identified these times as absorption times in conveniently defined auxiliary CTMCs, and their Laplace–Stieltjes transforms and different order moments have been computed algorithmically by means of a first-step analysis, while exploiting the quasi-birth-and-death structure of the infinitesimal generators associated with these processes. Moreover, the construction of the DP model as an extension of the IP model in §2 allows us not only to analyse the role played by phosphorylation events (see §3.3), but also to show how different reactions may be incorporated while adapting the matrix-analytic approach. A particular feature of this analytic approach is that it allows one to study the role played by each kinetic rate, by computing the partial derivatives of the descriptors under consideration with respect to the corresponding model parameters.

Our numerical results in §3 have considered the interaction between receptor VEGFR2 and bivalent ligand VEGF-A in human vascular endothelial cells. Our results indicate that phosphorylation, as an additional reaction, only seems to quantitatively affect the timescales for signalling (or phosphorylation), but does not qualitatively change the dynamics of the process. Moreover, by sequentially incorporating receptor synthesis and internalization dynamics, we found that intra-cellular receptor trafficking plays an important role in shifting the original signal (in terms of phosphorylated dimers) found on the cell surface into endosomal compartments, but where the dynamics of free receptors, monomers and non-phosphorylated dimers are well characterized with mathematical models exclusively describing the cell surface. These cell surface models allowed us as well to identify optimum ligand concentrations, which were qualitatively unchanged if synthesis and internalization events are included (figure 10).

**Figure 11.**
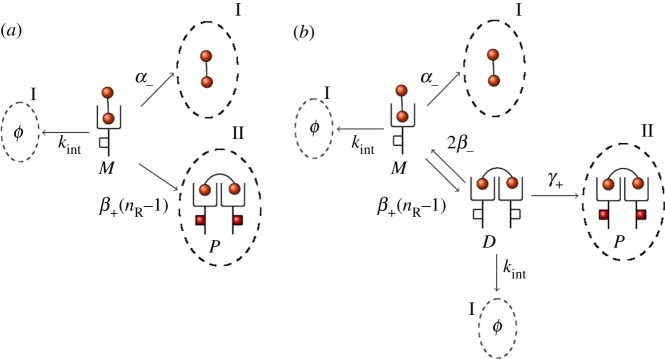
Individual bound monomer fate under (*a*) the extended IP model and (*b*) the extended DP model. Fate I: dissociation or internalization before signalling. Fate II: signalling before dissociation or internalization.

Our previous comments can be further illustrated by carrying out a single-molecule analysis; that is, by studying the fate of a bound monomer in the system. In particular, we consider a single ligand that has been captured by a receptor forming a bound monomer, and analyse the dynamics of this single complex, neglecting the effects due to other ligands or receptors in the system. Thus, we focus on the *fate* of this complex (phosphorylating or not before the bound monomer dissociates or internalizes), which depends on the kinetic rates, and is controlled by the stochastic processes illustrated in figure 11. We note that the original models (without internalization) can be obtained by setting *k*_int_ = 0 in figure 9, since in that case we also set *k*_syn_ = *n*_R_*k*_int_ = 0.

If we define
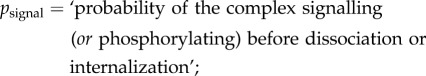
that is, the probability of Fate II. This probability can be computed as follows:

— IP model (instantaneous phosphorylation):
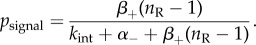
— DP model (delayed phosphorylation):
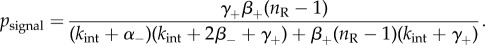

On the other hand, if we focus on the time to signalling and define
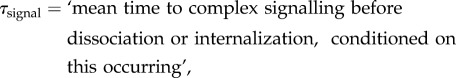
this conditioned mean time can be written as:

— IP model (instantaneous phosphorylation):
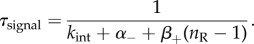
— DP model (delayed phosphorylation):
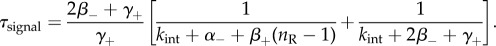

The values of *p*_signal_ and *τ*_signal_ are reported in table 4 for *k*_int_ ∈ {0, 2.8 × 10^−4^ s^−1^}. From these results, it seems clear that once a ligand is bound to a monomeric receptor, the probability to phosphorylate and, thus, to signal is almost one (for either model), when no internalization occurs. Internalization of complexes and delayed phosphorylation cannot decrease this probability on their own, and only when these two events are considered together, the single-molecule signalling probability of a monomer decreases approximately by 9%. However, the timescales to phosphorylate are mainly affected by the delayed phosphorylation. On the other hand, it might seem counterintuitive that the timescales for signal initiation are shorter when internalization takes place. We note here that these are conditioned times for signalling, that is, times conditioned on this signalling actually occurring. Thus, our results for *τ*_signal_ in table 4 should be interpreted as the fact that, if internalization can occur, only those monomers reaching dimerization and phosphorylation soon enough will initiate signalling before internalization takes place.

**Table 4.** Probability of a single monomer signalling (i.e. dimerizing and becoming phosphorylated, *p*_signal_) and conditioned time for this to occur (*τ*_signal_).

*k*_int_	model	*p*_signal_	*τ*_signal_
0	IP model	0.9874	9.5356 s
	DP model	0.9863	283.0799 s
2.8 × 10^−4^ s^−1^	IP model	0.9847	9.5095 s
	DP model	0.9137	265.1743 s

From a biological perspective, we note that the total number of VEGFR2s per cell varies according to other studies [30,39,47] and could be larger than the numbers used in our computations [42]. A larger number of VEGFR2 receptors on the cell surface would, however, only quantitatively change our results, and in particular a higher optimum ligand concentration threshold would be reported. The sensitivity analysis carried out for the descriptors enables us to show how the monomeric formation rate, *α*_+_, plays a crucial role in these models, with an effect which can be more than twice the effect of any other rate for some of the descriptors we have considered. Finally, the numerical results presented in §3 for the VEGF-A and VEGFR system have allowed us to quantify the effect of different ligand concentrations on the timescales to signalling, the late time behaviour of the system and the time course dynamics of the individual molecular species. Increasing ligand concentration decreases the times to reach any signalling threshold and increases the maximum potential signalling thresholds to be reached. However, high ligand concentrations can result in saturated scenarios, where the phosphorylation of bound dimers is reduced and monomeric bound complexes are enhanced.

The approach presented here could be, in principle, applied to other RTKs, most notably the EGFR, which is driving cellular proliferation in a variety of epithelial tumours. This receptor is of special relevance in clinical oncology, since a series of promising anti-EGFR small-molecule RTK inhibitors have already been designed. Unfortunately, drug resistance usually emerges during the course of treatment and it is important to understand the molecular mechanisms that underlie the development of such drug resistance, which may involve both the wild-type and mutant receptors [48]. Other RTKs of interest, for example, are those of the fibroblast growth factor receptor family, insulin receptor family and the leucocyte RTK family.

## Data accessibility

This article has no additional data.

## Appendix A. Notation

In this appendix, we set some standard notation to be used in the paper. First, *δ*_*i*,*j*_ represents Kronecker's delta; that is,

Given a set ,  represents its cardinality. Matrices and vectors are always given in bold, where **0**_*p*_ (**e**_*q*_) represents a column vector of zeros (ones) with dimension *p* (*q*). The symbol ^*T*^ represents the transposition operator and, for a matrix **A**(*θ*), we use the calculus notation

Finally, when a matrix depends on different parameters, **A**(*α*, *θ*), its first-order partial derivatives with respect to each parameter are given by **A**^(*α*)^(*α*, *θ*) and **A**^(*θ*)^(*α*, *θ*), respectively.

## Appendix B. Analysis of the IP model

The analysis carried out in this appendix requires the use of levels for the organization of the state space, Laplace–Stieltjes transforms, first-step arguments and auxiliary absorbing Markov chains. We first organize the state space , which contains

states, by levels (groups of states) as

where *L*(*k*) = {(*n*_1_, *n*_2_): *n*_2_ = *k*}, 0 ≤ *k* ≤ *n*_L_, so that *J*(*k*) = #*L*(*k*) = *n*_L_ − *k* + 1. That is, a level *L*(*k*) comprises all the possible states (*n*_1_, *n*_2_) of the process with a total number of *P* complexes equal to *k*. Moreover, we order these levels as

and states inside a level, *L*(*k*) = {(0, *k*), (1, *k*), …, (*n*_L_ − *k*, *k*)}, 0 ≤ *k* ≤ *n*_L_, are ordered as

Given the transitions of figure 2, it is clear that from a state (*n*_1_, *n*_2_) in level *L*(*n*_2_), the process can only move to states in the same level, *L*(*n*_2_), and to states in adjacent levels, *L*(*n*_2_ − 1) and *L*(*n*_2_ + 1). That is, if the state of the system is (*n*_1_, *n*_2_) (and then, the process is in level *L*(*n*_2_)), the only possible transitions are to (*n*_1_ − 1, *n*_2_) (if a bound monomer dissociates, in which case the process remains in level *L*(*n*_2_)), to (*n*_1_ + 1, *n*_2_) (if a bound monomer is formed, leaving the process in level *L*(*n*_2_)), to (*n*_1_ + 1, *n*_2_ − 1) (if a bound dimer dissociates, and the process then decreases to level *L*(*n*_2_ − 1)) or to (*n*_1_ − 1, *n*_2_ + 1) (if a bound dimer is created, increasing the level of the process to *L*(*n*_2_ + 1)).

The organization of , previously proposed, becomes crucial to obtain a convenient structure for the infinitesimal generator **Q** of , the matrix containing the transition rates in the Markov chain. In particular, the resulting **Q** is of the quasi-birth-and-death type [18] (tridiagonal by blocks structure)

rmB1

where sub-matrices **A** _*k*,*k*′_ contain the infinitesimal transition rates of the transitions from states in level *L*(*k*) to states in level *L*(*k*′), with *k*′ ∈ {*k* − 1, *k*, *k* + 1}. In particular, matrices **A** _*k*,*k*′_ in (B 1) are obtained from (2.1) and are as follows:

— For 1 ≤ *k* ≤ *n*_L_,
  where 0 ≤ *i* ≤ *n*_L_ − *k*, 0 ≤ *j* ≤ *n*_L_ − *k* + 1.
— For 0 ≤ *k* ≤ *n*_L_,
  where 0 ≤ *i* ≤ *n*_L_ − *k*, 0 ≤ *j* ≤ *n*_L_ − *k*.
— For 0 ≤ *k* ≤ *n*_L_ − 1,
  where 0 ≤ *i* ≤ *n*_L_ − *k*, 0 ≤ *j* ≤ *n*_L_ − *k* − 1.

We consider the time to obtain a threshold number, *N* > 0, of *P* complexes. In particular, given an initial state of the process (*n*_1_, *n*_2_), and a certain threshold *N* > 0, we consider the random variable

We observe that this time is 0 for *N* ≤ *n*_2_. In order to study this descriptor for *N* > *n*_2_, we make use of an auxiliary CTMC, , which depends on the threshold value *N*. We define  over the state space  with

where we denote , and where  is a macro-state that consists of all the states in the set ∪^*n*_L_^_*k*=*N*_*L*(*k*). Regarding the transitions of this auxiliary CTMC, we retain those transitions of  between states in , with  an absorbing state, so that once  enters , it does not leave this state. Transitions from states in level *L*(*N* − 1) to states in *L*(*N*) of the original process  become transitions from states in level *L*(*N* − 1) to the macro-state  in , where their infinitesimal transition rates are computed from the original ones as follows:

The process  can be seen as the process  until a number *N* of *P* complexes are formed. Then,  ends since  is an absorbing state for this auxiliary process. With  so defined, it is clear that the time taken to obtain a number *N* of *P* complexes in the original process  is equal to the time until absorption at  in the (absorbing) process , which is known to follow a continuous phase-type (PH) distribution (e.g. [9,18]). The analysis of the exact distribution of a continuous phase-type random variable is, in general, a difficult problem. In our case, it would imply obtaining the exponential matrix , where **T**(*N*) is a specific sub-matrix of the infinitesimal generator of . Here, we instead make use of the Laplace-Stieltjes transform of *T*_(*n*_1_,*n*_2__)__(*N*), which completely determines its distribution, and which allows us to obtain any *l*th-order moment *E*[*T*_(*n*_1_,*n*_2__)__(*N*)^*l*^]. We can also efficiently calculate the *l*th-order moment by using the (*l* − 1)th-order moment, proceeding recursively, with the computational effort devoted to obtaining inverses of square blocks **A**_*k*,*k*_, with dimension *J*(*k*) = *n*_L_ − *k* + 1. Again, the proposed organization of states is essential for the construction of an efficient algorithm. If we define the Laplace–Stieltjes transform of *T*_(*n*_1_,*n*_2__)__(*N*) as

then, the different *l*th-order moments of *T*_(*n*_1___,*n*_2__)__(*N*) can be obtained as

We can apply a first-step argument in order to obtain a system of linear equations for the Laplace–Stieltjes transforms *ϕ*^*N*^_(*n*_1__,*n*___2___)___(*z*)_, given a state . We can write down the equation

where from now on *A*_(_*_n_*_1_,*_n_*_2__)_ = 2*α*_+_(*n*_R_ − *n*_1_ − 2*n*_2_)(*n*_L_ − *n*_1_ − *n*_2_) + *α*_−_*n*_1_ + *β*_+_*n*_1_(*n*_R_ − *n*_1_ − 2*n*_2_) + 2*β*_−_*n*_2_. Equation (B 2) relates the Laplace–Stieltjes transforms of all the states in , so that a system of linear equations is obtained. If we organize the Laplace–Stieltjes transforms in vectors by levels as follows:

with **g**^*N*^_*k*_(*z*) = (*ϕ*^*N*^_(0,*k*)_(*z*), *ϕ*^*N*^_(1,*k*)_(*z*), *ϕ*^*N*^_(2,*k*)_(*z*), …, *ϕ*^*N*^_(*n*_L_ − *_k_*_,_*_k_*_)__(*z*))^T^, for 0 ≤ *k* ≤ *N* − 1, then the system given in (B 2) can be expressed in matrix form as

rmB3

with the matrix **A**^*N*^(*z*) given by

and the vector **a**^*N*^(*z*) = (**0**^T^_*J*(0)_, **0**^T^_*J*(1)_, …, **0**^T^_*J*(*N*−2)_, **a**_*N*−1_(*z*)^T^)^T^. Sub-matrices **A**_*k*,*k*′_(*z*) and sub-vector **a**_*N*−1_(*z*) in (B 3) are given by

— (**a**_*N*−1_(*z*))_*i*_ = *β*_+_*i*(*n*_R_ − *i* − 2(*N* − 1))/(*z* + *A*_(*i*,*N*−1)_), for 0 ≤ *i* ≤ *n*_L_ − *N* + 1.
— For 1 ≤ *k* ≤ *n*_L_,
  where 0 ≤ *i* ≤ *n*_L_ − *k*, 0 ≤ *j* ≤ *n*_L_ − *k* + 1.
— For 0 ≤ *k* ≤ *n*_L_,
  where 0 ≤ *i* ≤ *n*_L_ − *k*, 0 ≤ *j* ≤ *n*_L_ − *k*.
— For 0 ≤ *k* ≤ *n*_L_ − 1,
  where 0 ≤ *i* ≤ *n*_L_ − *k*, 0 ≤ *j* ≤ *n*_L_ − *k* − 1.

Exploiting the special block structure of **A**^*N*^(*z*) allows for an efficient solution of the system in (B 3), in a recursive manner through a specialized block-Gaussian elimination process, leading to algorithm 1 (Part 1). The calculation of the Laplace–Stieltjes transforms in algorithm 1 (Part 1) has its own merit, since it determines the distribution of the random variable under consideration. Moreover, the calculation of the distribution function of *T*_(*n*_1_,*n*_2__)__(*N*) by numerical inversion of the transform is possible, although computationally expensive, and is not developed here (e.g. [49]).

Once the Laplace–Stieltjes transforms are in hand, we can obtain the different *l*th-order moments by successive differentiation of the system in (B 3). In particular, we can write

rmB4

where **m**^*N*,(*l*)^ is the column vector containing the desired moments *E*[*T*_(*n*_1_,*n*_2__)__(*N*)^*l*^], for . We organize these moments in sub-vectors by levels as follows:

with **m**^*N*,(*l*)^_*k*_ = (*E*[*T*_(0,*k*)_(*N*)^*l*^], *E*[*T*_(1,*k*)_(*N*)^*l*^], *E*[*T*_(2,*k*)_(*N*)^*l*^], …, *E*[*T*_(*n*_L___− *k*, *k*)_(*N*)^*l*^])^T^, for 0 ≤ *k* ≤ *N* − 1. Note that the notation  is implicit in (B 4). That is, the moment of order *l* = 0 is the Laplace–Stieltjes transform for *z* = 0. Finally, the system in (B 4) is rewritten following the notation presented in appendix A:

rmB5

It is clear that the direct calculation of the inverse  involved in the solution of (B 5) can be avoided if working by levels and solving (B 5) in a similar way to algorithm 1 (Part 1). By starting with the known moment of order *p* = 0, we proceed recursively by calculating **m**^*N*,(*p*)^ from **m**^*N*,(*p*−1)^, until the desired order *p* = *l* is reached, leading to algorithm 1 (Part 2). Matrices **A**^*N*,(*p*)^(0) and **a**^*N*,(*p*)^(0) in (B 5) are given by

where expressions for **a**^(*p*)^_*N*−1_(0) and **A**^(*p*)^_*k*,*k*′_(0), for *p* ≥ 1, are as follows:

— (**a**^(*p*)^_*N*−1_(0))_*i*_ = ( − 1)^*p*^*p*!(*β*_+_*i*(*n*_R_ − *i* − 2(*N* − 1))/*A*^*p*+1^_(*i*,*N*−1)_), for 0 ≤ *i* ≤ *n*_L_ − *N* + 1.
— For 1 ≤ *k* ≤ *n*_L_, *p* ≥ 1,
  where 0 ≤ *i* ≤ *n*_L_ − *k*, 0 ≤ *j* ≤ *n*_L_ − *k* + 1.
— For 0 ≤ *k* ≤ *n*_L_, *p* ≥ 1,
  where 0 ≤ *i* ≤ *n*_L_ − *k*, 0 ≤ *j* ≤ *n*_L_ − *k*.
— For 0 ≤ *k* ≤ *n*_L_ − 1, *p* ≥ 1,
  where 0 ≤ *i* ≤ *n*_L_ − *k*, 0 ≤ *j* ≤ *n*_L_ − *k* − 1.

Finally, the late time behaviour of the process is given by the stationary probability distribution of the CTMC; that is, the probabilities

which do not depend on the initial state. We can store this probability distribution in a row vector *π* = (*π*_0_, *π*_2_, …, *π*_*n*_L__), where the row sub-vector *π*_*k*_ contains the ordered probabilities *π*_(*n*_1_,*n*_2_)_ for states in level *L*(*k*). Solving the system

and adapting arguments from Latouche & Ramaswami [9, ch. 10], we obtain algorithm 2. With ***π*** in hand, the long-term mean number of *M* and *P* complexes can be obtained as

## Appendix C. Analysis of the DP model

To study the descriptors described in §3.3, we again define levels in the state space, and arrange  in levels as follows:

where , for 0 ≤ *k* ≤ *n*_L_, so that

The three-dimensionality of our process implies that each level  may be split into sub-levels, as follows:

with , for 0 ≤ *r* ≤ *n*_L_ − *k*, 0 ≤ *k* ≤ *n*_L_, and *J*(*k*; *r*) = #*l*(*k*; *r*) = *n*_L_ − *r* − *k* + 1. That is,

and states in *l*(*k*; *r*) are ordered as indicated above.

The given order of states and the organization by levels and sub-levels yield an infinitesimal generator similar to (B 1), where quantities *J*(*k*) and matrices **A**_*k*,*k*′_ are replaced by  and , respectively. Matrix  contains the ordered infinitesimal transition rates corresponding to transitions from states in level  to states in level . Each matrix  is formed by sub-blocks **B**^*k*,*k*′^_*r*,*r*′_ which contain the infinitesimal transition rates corresponding to transitions from states in sub-level  to states in sub-level . We observe that the dimension of the matrix  is  , while the dimension of the sub-block **B**^*k*,*k*′^_*r*,*r*′_ inside  is *J*(*k*; *r*) × *J*(*k*′; *r*′) = (*n*_L_ − *r* − *k* + 1) × (*n*_L_ − *r*′ − *k*′ + 1). Expressions for these matrices are as follows:

— for 0 ≤ *k* ≤ *n*_L_
— for 0 ≤ *k* ≤ *n*_L_ − 1,
— for 1 ≤ *k* ≤ *n*_L_,

We note that, although we are omitting the dimensions of the matrices **0** for the ease of notation, the dimension of each matrix **0**, representing transitions from states in sub-level *l*(*k*; *r*) to states in sub-level *l*(*k*′; *r*′), is *J*(*k*; *r*) × *J*(*k*′; *r*′). The expressions for the matrices **B**^*k*,*k*′^_*r*,*r*′_ are given as follows:

— For 0 ≤ *r* ≤ *n*_L_ − *k*, 0 ≤ *k* ≤ *n*_L_,
  where 0 ≤ *i* ≤ *n*_L_ − *r* − *k*, 0 ≤ *j* ≤ *n*_L_ − *r* − *k*, and, from now on, *A*_(*i*,*r*,*k*)_ = 2*α*_+_(*n*_R_ − *i* − 2*r* − 2*k*)(*n*_L_ − *i* − *r* − *k*) + *α*_−_*i* + *β*_+_*i*(*n*_R_ − *i* − 2*r* − 2*k*) + 2*β*_−_*r* + *γ*_+_*r* + *γ*_−_*k*.
— For 0 ≤ *r* ≤ *n*_L_ − *k* − 1, 0 ≤ *k* ≤ *n*_L_,
  where 0 ≤ *i* ≤ *n*_L_ − *r* − *k*, 0 ≤ *j* ≤ *n*_L_ − *r* − *k* − 1.
— For 1 ≤ *r* ≤ *n*_L_ − *k*, 0 ≤ *k* ≤ *n*_L_,
  where 0 ≤ *i* ≤ *n*_L_ − *r* − *k*, 0 ≤ *j* ≤ *n*_L_ − *r* − *k* + 1.
— For 1 ≤ *r* ≤ *n*_L_ − *k*, 0 ≤ *k* ≤ *n*_L_ − 1,
  where 0 ≤ *i* ≤ *n*_L_ − *r* − *k*, 0 ≤ *j* ≤ *n*_L_ − *r* − *k*.
— For 0 ≤ *r* ≤ *n*_L_ − *k*, 1 ≤ *k* ≤ *n*_L_,
  where 0 ≤ *i* ≤ *n*_L_ − *r* − *k*, 0 ≤ *j* ≤ *n*_L_ − *r* − *k*.

For an initial state  and a number *N* > 0, we are also interested in the random variable

We omit *N* in the notation for convenience, and denote the random variable under study *T*_(*n*_1_,*n*_2_,*n*_3_)_. Again, this time is 0 for *N* ≤ *n*_3_. For *N* > *n*_3_, we follow an argument similar to that of appendix B, so that *T*_(*n*_1_,*n*_2_,*n*_3_)_ is studied as an absorption time in a suitable auxiliary process.

In order to obtain the different *l*th-order moments in an efficient way, we define the Laplace–Stieltjes transform of *T*_(*n*_1_,*n*_2_,*n*_3_)_ as

and the different *l*th-order moments of *T*_(*n*_1_,*n*_2_,*n*_3_)_ can be obtained as

By a first-step argument (omitted here since it is analogous to (B 2)), we obtain the system

rmC1

where the Laplace–Stieltjes transforms are stored in vectors , following the order given by the levels and sub-levels, and where the expressions for matrices  and  are omitted for brevity. By successive differentiation of the system in (C 1), we obtain the different *l*th-order moments *E*[*T*^*l*^_(*n*_1_,*n*_2_,*n*_3_)_] through an adapted version of algorithm 1, with the *l*th-order moments stored in the vectors . We note that in the adapted version of algorithm 1 to solve (C 1), which is omitted, we need to deal with inverses of matrices with dimension . The complexity of transitions between states does not allow us to gain further efficiency in our algorithms by working with inverses of matrices with the dimensions of the given sub-levels. However, in the special case *γ*_−_ = 0, that is, when de-phosphorylation is neglected, it is possible to improve the procedures so that the highest computational effort is placed on inverting matrices with the dimensions of sub-levels instead of levels, which would yield an algorithm 3, not described here.

Finally, we focus on the stationary distribution of the process, that is, the probabilities

which do not depend on the initial state. Similar arguments to those considered in appendix B allow us to obtain the stationary distribution in a row vector , where , and where row sub-vectors  contain, in an ordered manner, steady-state probabilities of states in sub-levels *l*(*k*; *r*). An adapted version of algorithm 2 can be obtained, where the matrices **A**_*j*,*j*′_, in (B 1), would be now replaced by the matrices  previously defined. Once these vectors are in hand, it is clear that

## Appendix D. Local sensitivity analysis for the stochastic descriptors

The objective of this appendix is to develop a local sensitivity analysis to understand the effect that each of the (association, dissociation, phosphorylation or de-phosphorylation) rates (*α*_+_, *α*_−_, *β*_+_, *β*_−_, *γ*_+_ and *γ*_−_) has on the stochastic descriptors introduced (appendices B and C) for the DP and the IP models, in a given neighbourhood of parameter space. This selected neighbourhood of parameter space may be obtained from a parameter estimation of *in vitro* and *in silico* experiments, as shown in §3.1. Our aim then is to obtain the partial derivatives of our descriptors with respect to each parameter, so that these derivatives provide a measure of the effect of a perturbation of the parameters on the descriptors.

Sensitivity analysis for CTMC with absorbing states has been recently developed [22]. Although the Markov chains considered in our models are, in general, non-absorbing, the arguments in Caswell [22] can be clearly generalized to the CTMCs considered here; see Gómez-Corral & López-García [23] for how to adapt these arguments to structured Markov processes such as the ones considered in this study. We briefly explain how to adapt them in what follows, while keeping the spirit of the matrix-analytic approach applied in previous sections.

We note that the descriptors of §3.3 are analysed in appendices B and C by following a matrix-oriented methodology, where algorithms 1 and 2 depend on the use of matrices **A**_*k*,*k*′_(*z*), **H**_*k*_(*z*) and inverses **H**^−1^_*k*_(*z*), which are matrices that clearly depend on parameters (*α*_+_, *α*_−_, *β*_+_, *β*_−_, *γ*_+_, *γ*_−_). Thus, when carrying out a local sensitivity analysis (in terms of partial derivatives) by adapting arguments of Caswell [22], one would need to compute the element-by-element partial derivatives of these matrices with respect to the parameters of interest. We note that given any matrix **B**_*m*×*n*_(***θ***), that depends on some parameters vector ***θ***, we denote by **B**^(*θ*_*i*_)^(***θ***) its element-by-element derivative with respect to *θ*_*i*_ ∈ ***θ***. It is possible to calculate the derivative of its inverse, **B**^−1^(***θ***), with respect to *θ*_*i*_ from **B**^(*θ*_*i*_)^(***θ***) as [50,51]

We make use of this and other basic matrix calculus properties, as discussed in Caswell [22], to obtain algorithms 1S and 2S, which are given below, and can be obtained by sequentially differentiating all matrices in algorithms 1 and 2, respectively. Finally, the explicit expressions for matrices in these algorithms, consisting of the element-by-element partial derivative of the matrices defined in appendices B and C, with respect to any parameter, *θ*_*i*_ ∈ {*α*_+_, *α*_−_, *β*_+_, *β*_−_, *γ*_+_, *γ*_−_}, are not reported here.

It is clear that, since our descriptors are stored in the vectors **m**^*N*,(*l*)^,  (time to reach a threshold number of *P* complexes in the IP and DP models, respectively) and the quantities *π*_*j*_ and  (mean number of *j* complexes in steady state in the IP model (*j* ∈ {*M*, *P*}) and the DP model (*j* ∈ {*M*, *D*, *P*}), respectively), the objective in algorithms 1S and 2S is to obtain the derivative vectors **m**^*N*,(*l*,*θ*_*i*_^^)^, , ***π***^(*θ*_*i*_^^)^ and . The first two vectors contain the derivatives of the *l*th-order moments of the time to reach a given threshold number of *P* complexes, and the last two yield the derivatives of the quantities *π*_*j*_ and , with respect to each rate *θ*_*i*_ ∈ {*α*_+_, *α*_−_, *β*_+_, *β*_−_, *γ*_+_, *γ*_−_}.

We point out that **m**^*N*,(*r*,*θ*_*i*_)^_*k*_ and **A**^(*r*,*θ*_*i*_)^_*k*,*k*′_(0) in algorithm 1S, which corresponds to the model with instantaneous phosphorylation, represent the derivatives of **m**^*N*,(*r*)^_*k*_ and **A**^(*r*)^_*k*,*k*′_(0), respectively, with respect *θ*_*i*_, for *θ*_*i*_ ∈ {*α*_+_, *α*_−_, *β*_+_, *β*_−_}.
